# Nonfibrillar Dutch mutant amyloid beta (Aß) aggregates (oligomers) are associated with aging‐related synaptic dysfunction

**DOI:** 10.1002/alz.084937

**Published:** 2025-01-03

**Authors:** Sam E Gandy, Emilie L Castranio, Merina Varghese, Elentina K Argyrousi, Kuldeep Tripathi, Linda Söderberg, Erin Bresnahan, David Lerner, Francesca Garretti, Hong Zhang, Jonathan Van de Loo, Ronan Talty, Charles G Glabe, Efrat Levy, Minghui Wang, Bin Zhang, Lars Lannfelt, William D Lubell, Brigitte Guerin, Shai Rahimipour, Dara Dickstein, Ottavio Arancio, Michelle E. Ehrlich

**Affiliations:** ^1^ Icahn School of Medicine at Mount Sinai, New York, NY USA; ^2^ Columbia University, New York, NY USA; ^3^ Bar‐Ilan University, Raman Gat Israel; ^4^ BioArctic AB, Stockholm Sweden; ^5^ Icahn School of Medicine at Mount Sinai, NEW YORK, NY USA; ^6^ Icahn School of Medicine Mount Sinai, New york, NY USA; ^7^ Yale University, New Haven, CT USA; ^8^ University of California Irvine, Irvine, CA USA; ^9^ Nathan S. Kline Institute for Psychiatric Research, Orangeburg, NY USA; ^10^ New York University Grossman School of Medicine, New York, NY USA; ^11^ Icahn Institute for Data Science and Genomic Technology, New York, NY USA; ^12^ Mount Sinai Center for Transformative Disease Modeling, New York, NY USA; ^13^ Ronald M. Loeb Center for Alzheimer’s Disease, New York, NY USA; ^14^ BioArctic, Stockholm Sweden; ^15^ University of Montreal, Montreal, QC Canada; ^16^ University of Sherbrooke, Sherbrooke, QC Canada; ^17^ Bar Ilan University, Raman Gat Israel; ^18^ Uniformed Services University of Health Science, Bethesda, MD USA

## Abstract

**Background:**

Clinicopathological studies of Alzheimer’s disease (AD) have demonstrated that synaptic or neuronal loss and clinical cognitive decline do not reliably correlate with fibrillar amyloid burden. We created a transgenic mouse model overexpressing Dutch (E693Q) mutant human amyloid precursor protein (*APP*) driven by the pan‐neuronal *Thy1* promoter. Accumulation of *APP* carboxyl‐terminal fragments was observed in the brains of these mice, which develop an impaired learning phenotype directly proportional to brain oAβ levels.

**Method:**

Male and female TgAPP^E693Q^ mice and wildtype controls were compared using learning behavioral studies, immunocytochemistry, transmission electron microscopy, electrophysiology, protofibril‐specific assays, and single cell RNA sequencing.

**Result:**

Brain levels of nonfibrillar oAβ in Dutch mice were shown to increase aging‐dependently using A11 immunocytochemistry and FITC‐cyclic peptide (FITC‐CP‐2) microscopy. Two assays excluded the presence of protofibrils. Electrophysiological characterization of hippocampal synapses in Dutch and wildtype mice at ∼7 and ∼11 months revealed no change in basal excitatory transmission, consistent with normal density and morphology of mGluR2/3+ synapses in hippocampal CA1 of the same mice. One exception was increased postsynaptic density in non‐perforated mGluR‐2/3+ synapses in the Dutch mice. Functional characterization of the presynaptic terminal showed abnormalities in post‐tetanic potentiation, synaptic fatigue, and synaptic replenishment after depletion in Dutch mice. Single cell RNA‐seq to elucidate cell‐type specific transcriptional responses to oAβ revealed altered transcriptional profiles in multiple cell types. Unexpectedly, no obvious differences existed between profiles of microglia from Dutch compared to those from wildtype mice. Excitatory neurons showed the most altered profile which was associated with ‘protein translation’ and ‘oxidative phosphorylation’. Ultrastructural analysis of presynaptic mitochondria at excitatory synapses revealed fewer mitochondria in the presynaptic terminals of Dutch mice.

**Conclusion:**

The profound learning behavior deficits in Dutch mice are associated with presynaptic functional deficits and mitochondrial abnormalities in excitatory neurons of the hippocampus. Nonfibrillar oAβ deposits were revealed by co‐localization of A11 immunoreactivity with FITC‐CP‐2 microscopy. Mice accumulating only oAβ may be especially useful for further characterization of the oligomer‐specific cyclic azaglycine PET tracer Lys (^64^Cu/NOTA)^1^]‐CP‐7 that shows robust PET signal from 44‐day‐old presymptomatic 5xFAD mice [Habashi, M. *et al. Proc. Natl. Acad. Sci. U.S.A*. 2022].

